# Contributions of mean temperature and temperature variation to population stability and community diversity

**DOI:** 10.1002/ece3.8665

**Published:** 2022-02-22

**Authors:** Edd Hammill, Riley Dart

**Affiliations:** ^1^ 4606 Department of Watershed Sciences and the Ecology Center Utah State University Logan Utah 84341 USA

**Keywords:** community ecology, diversity, protist microcosms, stability, temperature change

## Abstract

Future climate changes are predicted to not only increase global temperatures but also alter temporal variation in temperature. As thermal tolerances form an important component of a species’ niche, changes to the temperature regime have the capacity to negatively impact species, and therefore, the diversity of the communities they inhabit. In this study, we used protist microcosms to assess how mean temperature, as well as temporal variation in temperature, affected diversity. Communities consisted of seven species in a multitrophic food web. Each ecosystem was inoculated with the same abundances of each species at the start of the experiment, and species densities, Hill's numbers (based on Shannon diversity), the number of extinctions, and the probability the microcosm contained predators were all calculated at the end of the experiment. To assess how mean temperature and temperature fluctuations affect stability, we also measured population densities through time. We found that increased temporal variation in temperature increased final densities, increased Hill's numbers (at low mean temperatures), decreased rates of extinctions, and increased the probability that predators survived till the end of the experiment. Mean temperatures did not significantly affect either the number of extinctions or the probability of predators, but did reduce the positive effect of increased temporal variation in temperature on overall diversity. Our results indicate that climatic changes have the potential to impact the composition of ecological communities by altering multiple components of temperature regimes. However, given that some climate forecasts are predicting increased mean temperatures and reduced variability, our finding that increased mean temperature and reduced temporal variation are both generally associated with negative consequences is somewhat concerning.

## Graphical abstract

Climatic changes are predicted to alter mean temperatures and fluctuations in temperature. Here, we show that increased mean temperatures are generally associated with negative impacts on biodiversity. However, these negative impacts can be partially reduced if fluctuations in temperature are maintained.

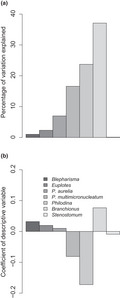

## 1 INTRODUCTION

Ongoing and future climatic changes are predicted to not only change mean temperatures (Masson‐Delmotte, 2018) but also levels of temporal fluctuation in temperature (Bathiany et al., 2018). Previous studies have suggested that temporal variation in temperature, a measure that describes how temperatures within a single location vary daily or seasonally, may be altered by climate change (Di Cecco & Gouhier, 2018). These variations in temporal variation may occur in either direction, leading to reduced variation (i.e., increased autocorrelation (Di Cecco & Gouhier, 2018)), or higher levels of variation with more extreme climatic events (Baker et al., 2018; Bathiany et al., 2018). Given that a changing climate may alter temporal variation either up or down, it is critical to understand how changes in mean temperature, as well as temporal variation, affect ecological communities (Thompson et al., 2013). Changes to average temperatures have been shown to impact ecological communities (Greig et al., 2012; Kratina et al., 2012); however, the relative impact of changes to mean temperature compared to changes in temporal variation to changes in diversity, extinction, and predator persistence remains relatively unexplored.

The persistence of species within a community is influenced by population sizes (Fagan et al., 2001) and population fluctuations (Hung et al., 2014; Ovaskainen & Meerson, 2010). Small populations are susceptible to demographic stochasticity, catastrophic impacts, inbreeding, and reduced ability to adapt to environment changes, all of which can lead to extinction (Purvis et al., 2000). Populations may fluctuate due to seasonal environmental changes (Krebs, 2013; Pinheiro et al., 2002), predator–prey interactions (Hammill et al., 2010; May & McLean, 2007; Tollrian et al., 2015), decadal oscillations (Ballard et al., 2003), changes in habitat distributions (Runge et al., 2014), and stochastic, episodic events (Dixon et al., 1999). High magnitude fluctuations in population size increase the probability that population sizes will drop to levels where the threats associated with small populations come into play, increasing extinction risk. The relationships between population size, fluctuations, and extinction risk make it critical to understand how changes to temperature regime influence population trajectories.

Fluctuations in population sizes scale up to affect the composition of whole ecological communities (Donohue et al., 2013). The overall diversity of ecological communities is determined by the number of species within the system (species richness) and the relative abundances of those species (species evenness). Species richness and evenness can be combined together to produce indices of diversity, such as the Shannon index (Shannon, 1948). Measuring diversity using indices of this form allow the teasing apart of differences among communities, as they not only account for the number of species in a community but also the abundance of species relative to others. Changes in the relative abundance of species form a critical part of the overall species diversity. In one community where species X is at very low densities, it may perform an ecological function to a far lesser degree than in another community where it is highly abundant (Stuart‐Smith et al., 2013). Hence, although there may be no change in species richness between the two communities, the difference in the relative abundance of species X may alter overall diversity through changes in relative abundances (i.e., species evenness), with implications for ecosystem functions. It is therefore important to incorporate aspects of species evenness within diversity measures to account for these potentially ecologically important changes in relative abundance. While the use of the Shannon diversity index represents an improvement over species richness as it accounts for species richness, it is also somewhat flawed in that as species are lost from a community the Shannon index does not reduce proportionally (Roswell et al., 2021). The use of Hill's numbers based on Shannon entropies represents an improvement over the Shannon diversity index by producing a metric that better scales with species loss (Roswell et al., 2021).

The persistence of species within communities is a major contributor to diversity, as even if a species’ abundance is reduced to very low levels, its continued persistence in a community maintains the potential for its population to increase in the future (Hammill, Kratina, et al., 2015). However, if a species is driven to extinction, in the absence of rescue effects, there is no potential for it to return in the future, and the functions performed exclusively by the species are lost (Petchey et al., 2004). Even at low densities, predators in particular have the capacity to influence the structure of ecological communities through top‐down effects (Hammill, Atwood, et al., 2015; Shurin et al., 2012; Terborgh, 2015). The important impact of predators warrant special attention in analyses of diversity, as changes to diversity metrics based on richness and evenness may fail to detect changes in the predator guild due to their relatively low abundances (and hence small contribution to overall diversity indices).

Experimental protist microcosms have become important tools for research into community and population ecology due to their short generation times and ease of manipulation (Altermatt et al., 2015; Benton et al., 2007). The ease of conducting multigenerational experiments has resulted in protist microcosms being previously used to investigate the relationships between changes in temperature and diversity (Petchey et al., 1999) and the factors that impact the persistence of species (Forbes & Hammill, 2013; Hammill et al., 2010; Lawler & Morin, 1993). As microcosms can be maintained for multiple generations under precise environmental conditions, they can be used to investigate factors that influence diversity at the ecosystem scale (Hammill & Clements, 2020; Holyoak & Lawler, 1997). Although they may be physically small in size, protist microcosms are able to contain a diverse range of species that are involved in multiple trophic and competitive interactions (Hammill & Clements, 2020). This diversity of species, trophic guilds, and interspecific interactions means that protist microcosms are able to replicate many of the processes that determine the behavior of natural ecological systems (Benton et al., 2007).

In this study, we quantify how changes in mean temperature, together with alterations in temporal variation in temperature affect population densities, stability, diversity, extinctions, and the persistence of predators using protist microcosms. Understanding the impact of temporal variation on diversity is important as previous investigations have illustrated that climate variability may pose a greater threat to ecosystems than changes in mean temperature (Thornton et al., 2014; Vasseur et al., 2014) and that certain measures of diversity are affected to a greater extent than others by variations in temperature (Hammill, Hawkins, et al., 2018). Given the possibility that climate change could alter patterns of temporal variation as well as mean temperatures (Di Cecco & Gouhier, 2018), it becomes imperative to understand the impact of different components of the temperature regime. Our goal was to understand the relative contributions to diversity that are made by changes in mean temperature as well as temporal variation temperature. We are specifically testing the hypotheses that increased temperature will increase extinctions and reduce diversity, while increased temporal variation in temperature will increase species diversity. We hypothesize that higher extinctions and reduced diversity will occur with increasing mean temperatures as higher temperatures will increase the strength of predator–prey interactions (Robertson & Hammill, 2021), and food webs containing strong interactions are associated with reduced levels of diversity (McCann, 2000). In addition, we hypothesize that increased temporal variation in temperature will increase diversity based on previous investigations into the effects of spatial variation showing that increased environmental variation is associated with increased diversity (Kraft et al., 2008; Longhi & Beisner, 2010; Tamme et al., 2010). The proposed mechanism for variation increasing diversity is based on niche partitioning, with heterogeneous locations providing a range of conditions for species with different niches (Brown et al., 2013). While our current study is not exactly analogous to these previous works as our fluctuations are temporal rather than spatial, changes in temperature through time may mean that different species are better suited at different times, reducing the probability a single species becomes dominant (Jiang & Morin, 2007). However, we would also like to highlight that high fluctuations in temperature may lead to extinctions and a reduction in diversity if the magnitude of fluctuations is so great that species are unable to cope with acute exposure to temperature extremes (Duarte et al., 2012).

## 2 METHODS

The experiment was conducted using protist communities housed in 150 ml of media in 200‐ml glass jars. Media consisted of 0.4 g L^−1^ protist pellets (no. 13‐2360; Carolina Biological Supply, Burlington, NC, USA), that were crushed in a pestle and mortar before being dissolved in Purelife™ mineral water (Nestle, USA). All media was sterilized in an autoclave prior to use. Each experimental microcosm was inoculated on day zero with 200 *Paramecium aurelia* (ciliate protist), 200 *Paramecium multimicronucleatum* (ciliate protist), 200 *Euplotes* (ciliate protist), 200 *Blepharisma* (ciliate protist), 50 *Philodina* rotifers, 50 *Branchionus* rotifers, and 10 *Stenostomum* flatworms. These inoculation densities were based on previous studies using the same species (Forbes & Hammill, 2013) and are high enough to reduce the risk of stochastic extinctions at the start of the experiment, but still well below carrying capacities. The community was sustained on a basal bacterial resource consisting of *Serratia* and *Bacillus subtilis* that were inoculated into the microcosms 24 h prior to the rest of the community and also contained microflagellates that were inoculated with the other protists. While these microflagellates have the potential to impact community processes within the microcosms, we classified them as part of the basal resource (which was specifically maintained to be in excess) and hence were not quantified. Within the communities, *Stenostomum* represent the highest trophic level, capable of consuming *Paramecium*, *Euplotes*, and *Blepharisma*. All protists and rotifers consume the same basal resource and so can be considered competitors. The food web therefore contains multiple predatory and competitive interactions (Hammill & Clements, 2020).

Temperature treatments were maintained using a series of water baths constructed from 36‐gallon aquaria maintained at the correct temperatures using Marineland Precision© 200w aquarium heaters (Marineland, Blacksburg, Virginia, USA). Temperatures were monitored using Hobo© Pro Temp V2 temperature loggers (Onset, Massachusetts, USA). Water bath temperatures were set at 16°C, 18°C, 20°C, 22°C, and 24°C and were maintained at ±0.3°C of these temperatures throughout the experiment. Stock cultures of each species were maintained at a constant temperature of 20°C for over 120 days prior to use, and 4°C is within the range of climate change predicted by 2100 but below the highest estimate of 5.4°C (Masson‐Delmotte et al., 2021). We therefore selected these as our experimental temperatures as the mean temperature was analogous to “current conditions”; the highest temperature was toward the upper end of that predicted by the end of the century, and the lower end represented the inverse of this prediction. The range of temperatures selected in the experiment was therefore relevant to predicted global changes and produced a good range (8°C) over which to observe changes. In addition, several of the species we are using have been observed to grow and feed across our temperature range (Robertson & Hammill, 2021), and the range is well within the critical limit of our top predator (Gamo & Noreña‐Janssen, 1998). The site from where several of our species were collected (First Dam, Logan River, UT, 41.7418, −111.7919) experiences annual temperatures between freezing and greater than 24°C, suggesting that all species should tolerate the thermal range. In total, the experiment consisted of 32 microcosms arranged in nine different experimental treatments. The first three treatments consisted of microcosms held constant at each of 18°C, 20°C, 22°C (*n* = 4 per treatment, mean = 18^o^C, 20°C, 22°C, SD = 0). The “small change” treatment consisted of four microcosms that were moved 2°C every 48 h between the 18°C, 20°C, and 22°C water baths (*n* = 4, mean = 20°C, SD = 1.43°C). In the “large change” treatment (*n* = 4, mean = 20°C, SD = 2.48°C), microcosms where moved 2°C every 48 h among all the water baths (i.e., 16°C→18°C→20°C→22°C→24°C→22°C→20°C…). In the “large change fast” treatment (*n* = 4, mean = 20°C, SD = 2.87°C), microcosms were moved 4°C every 48 h among the 16°C, 20°C, and 24°C water baths. In the “random” treatment (*n* = 8, mean = 19°C–21.2°C, SD = 2.45°C–2.92°C), each microcosm was moved on a different, randomly assigned path among the different water baths. The paths were generated using a random number generator, and there were no constraints on the maximum difference in temperature a microcosm could be moved. These “random” replicates meant that for every value of mean temperature, we had a range of fluctuations in temperature through time. While our experimental design is not completely balanced (more treatments have a mean temperature value of 20 than any other temperature), the variation stemming from the “random” replicates allows independent assessment of the changes to mean temperature and temporal variation in temperature. Across all treatments, the mean temperature and the temperature standard error were calculated at the end of the experiment. In total, the experiment ran for 24 days.

Each microcosm was sampled every 48 h. On each sampling occasion, the media in each microcosm was well mixed by pipetting, before 10ml of media was removed placed in a Bogorov counting chamber. As *Stenosomum* represented the top predators in the system and were also the least abundant, we used the entire 10ml sample to quantify their densities. A 1ml subsample of the 10ml sample was then used to quantify densities of the remaining species. All analyses were performed on species densities, designated as the number of individuals per ml. Following the completion of sampling, we added 10 ml of fresh protist media to each of the microcosms to maintain a constant volume throughout the experiment and to ensure some nutrients were continuously present. At the end of the experiment, we analyzed the whole 200ml community to check for the presence of rare species.

We analyzed the impact of changes in mean temperature and temporal variation in temperature on multiple components of diversity. At the population level, we investigated how changes to the temperature regime impacted final densities of each species, and the change in population densities through time, measured as the coefficient of variation (standard deviation divided by the mean). At the community level, we analyzed final diversity of each ecosystem using Hill's numbers based on Shannon entropy (Chao et al., 2014; Hill, 1973; Shannon, 1948), the number of extinctions observed in each ecosystem, and the probability that ecosystems contained predators. In this experiment, we did not define “extinction” as a species simply dropping below the detection limit (1ml sample for all species except *Stenostomum*). While nondetections (i.e., population sizes of zero) were included in the quantification of populations through time, a species was only classified as extinct if it was not detected in the final checking of the whole 200 ml community. We specifically compared whether or not each community contained top predators (*Stenostomum*) as this species has been shown to be able to significantly alter the structure of communities (Forbes & Hammill, 2013) and drive species to extinction (Hammill, Kratina, et al., 2015).

### 2.1 Statistical analyses

Data were analyzed using linear models with polynomial terms (allowing curved relationships) to determine whether our response variables had any effect on population metrics or community composition. Across all analyses, we chose not only to focus solely on P‐values but also highlight the amount of variation associated with each descriptive variable. To determine the relative importance of each descriptive variable in the models, the *R* ^2^ value for the whole model was decomposed using the value “lmg” from the “calc.relimp” function in the “relaimpo” package (Grömping, 2006). The lmg metric partitions the *R* ^2^ value into non‐negative portions for each variable. The sum of these portions then forms the total *R* ^2^ (Lindeman et al., 1980). However, the order in which the descriptive variables are initially added can influence their relative importance, so the lmg metric quantifies the relative importance of each variable averaged over every order in which they can be added to the linear model (Grömping, 2006; Johnson & LeBreton, 2004).

## 3 RESULTS

**FIGURE 1:**
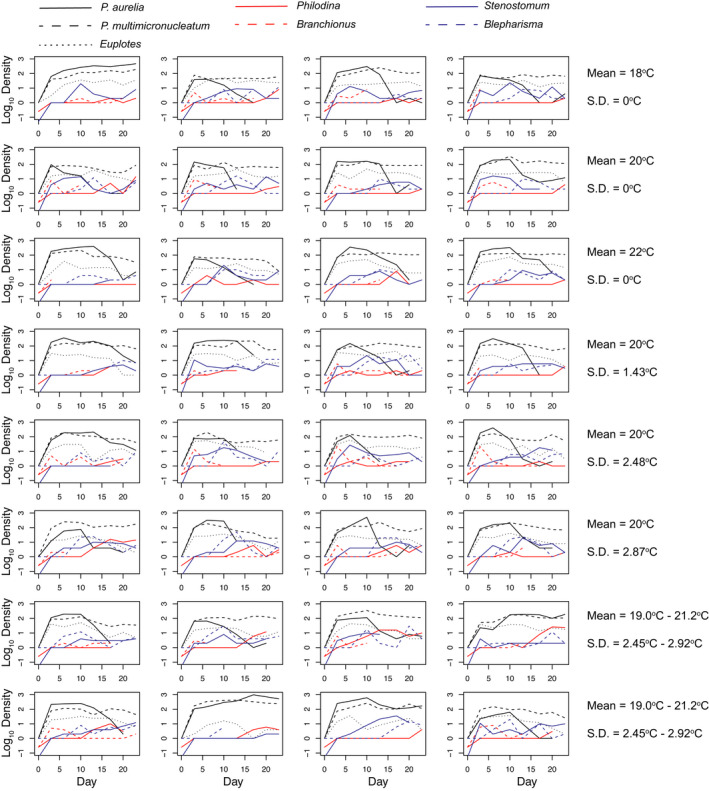
Log_10_ population densities of each species through time for the 32 replicate communities used in the experiment. Line end points indicate when a species was no longer detected in the sample, that is, an extinction

Population dynamics for all species are shown in Figure 1. Across all temperature regimes, we observed that multiple species in multiple trophic levels were observed to go extinct (Figure 1). Generally, we found that the bacterivorous ciliate protozoa (*Paramecium* and *Euplotes*) had the highest population densities, while the two rotifers were the lowest, with *Branchionus* rotifers also showing the highest number of extinctions (Figure 1). The ciliate species (*Paramecium*, *Euplotes*, and *Blepharisma*) generally appeared to show rapid exponential growth that plateaued and then often declined as predatory *Stenostomum* or other competitors increased (Figure 1).

**FIGURE 2:**
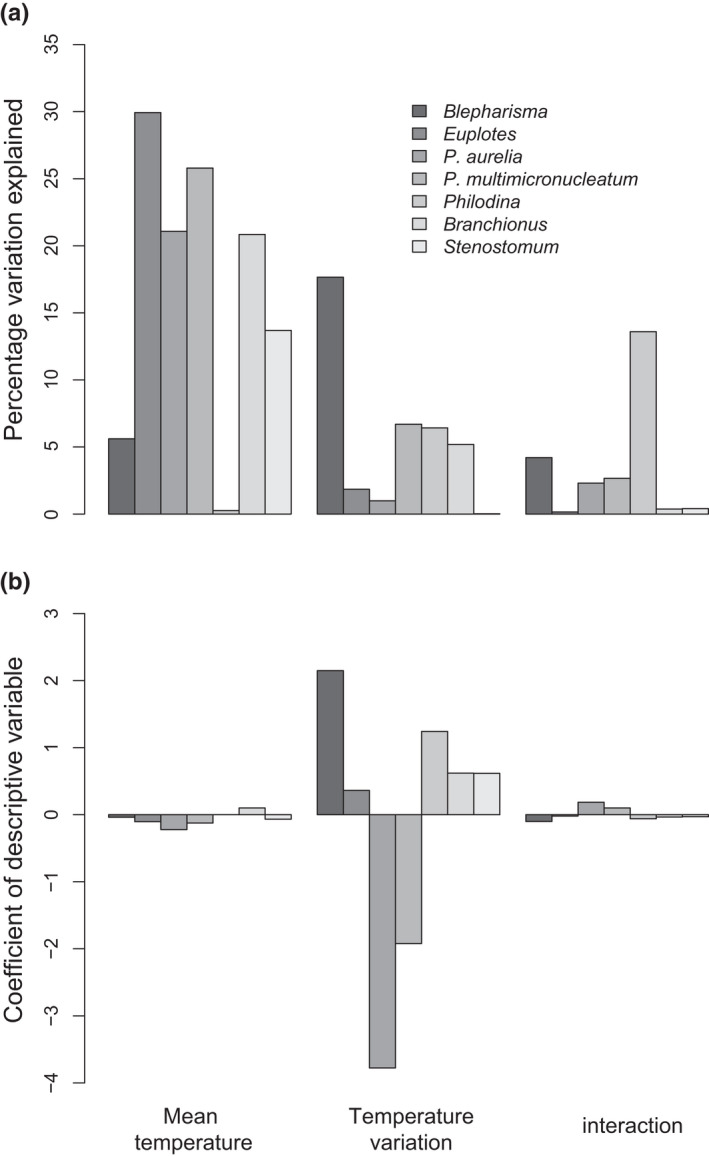
Relationships between different aspects of the temperature regime and final densities. (a) the amount of variation in final density explained by mean temperature, temporal variation in temperature, and an interaction between the two. (b) the slope value of the relationship between different aspects of the temperature regime and final densities. Values greater than 0 indicate a positive relationship between final densities and either mean temperature variation in temperature, or an interaction between the two

We found that final densities were significantly affected by interactions between taxa identity and each of mean temperature and temporal variation in temperature (all *p* < .05). Given the significant interactions, and difficulties associated with partitioning the variation for categorical variables, we split the data on the basis of taxa and re‐ran the analyses. We found that final densities for only one taxon (*Philodina* rotifers) were significantly affected by an interaction between mean temperature and temporal variation in temperature (Figure 2), and the amount of variation in final density explained by the interaction was generally low (0.15%–13.59%, Figure 1a). Across all taxa, changes in mean temperature accounted for a greater amount of change in final densities (16.74% ± 4.07%, Figure 2a) compared to temporal variation in temperature (5.55% ± 2.26%, Figure 2a). We found that higher mean temperatures were generally associated with reduced final densities (negative coefficients, Figure 1b), while changes in temporal variation were associated with increased final densities in all taxa except the two *Paramecium* species (Figure 2b).

**FIGURE 3:**
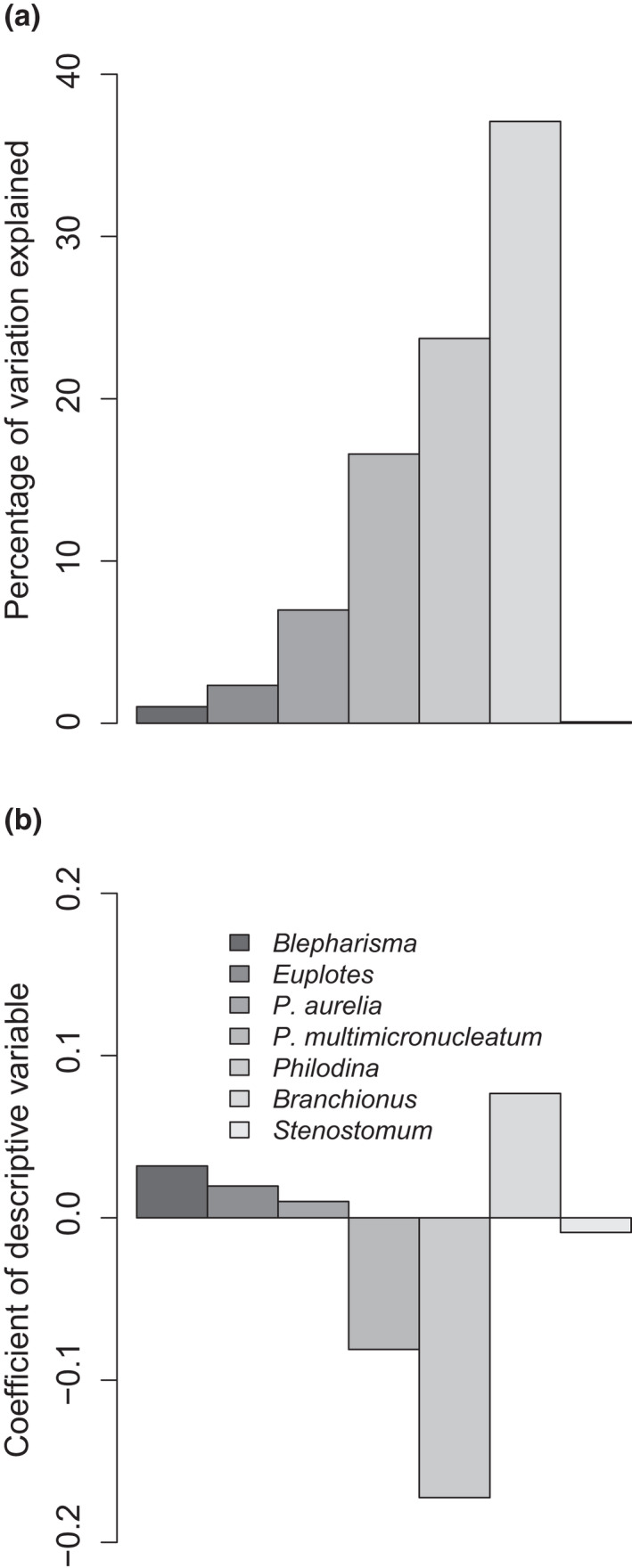
Effects of change in mean temperature on the coefficient of variation (CV) of population density through time for each species. (a) the amount of variation in population density CV explained by mean temperature (b) The slope of the relationship between population density CV and mean temperature, values greater than 0 indicate a positive relationship. Changes in temporal variation in temperature had no significant effect on population size CV

The coefficients of variation of population size through time only differed significantly with an interaction between taxa identity and mean temperature (*F*_(6, 182)_ = 5.13, *p* < .001). Due to this significant interaction, we split the data on the basis of species and ran individual models. We found that the coefficient of variation of density was only significantly affected by changes in mean temperature for three of the taxa (*P*. *multimicronucleatum*, *Philodina*, and *Branchionus*, Figure 3). For these three taxa, the amount of variation in population density CV explained by mean temperature was relatively high, ranging between 16.49% and 37.09% (Figure 3a), with a positive relationship between CV and temperature observed for *P*. *multimicronucleatum* and *Philodina* (indicating less stability at high temperatures) and a negative relationship observed for *Branchionus*. Changes in temperature variation were not associated with significant changes in density CV through time for any species (all *p* > .05).

**FIGURE 4:**
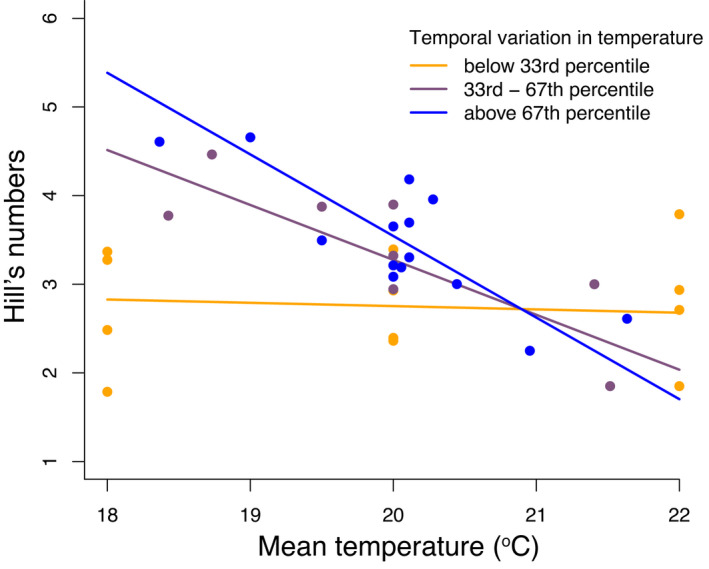
Hill's numbers (based on Shannon diversity) at the end of the experiment were significantly affected by an interaction between mean temperature and variation in temperature. Panel shows how the relationship between mean temperature and diversity is affected by changes in mean temperature. Lines represent the model fit; the points show the raw data. Highest diversities were observed at high levels of temporal variation in temperature (above 66^th^ percentile) when mean temperatures were low

**FIGURE 5:**
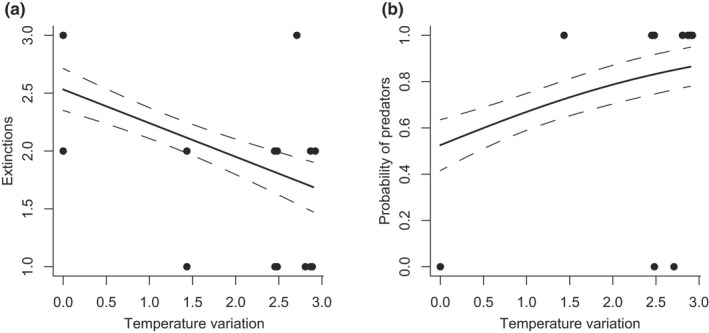
Changes in the level of temporal variation in temperature affect (a) the number of extinctions observed in the communities and (b) the likelihood a community contained predators. Changes in mean temperature did not affect either metric. Central solid lines represent the relationship, dashed lines denote standard errors, and points represent the raw data

With respect to changes in overall communities, we found that Hill's numbers at the end of the experiment were significantly altered by an interaction between mean temperature and variation in temperature (*F*_(1, 28)_ = 5.98, *p* = .019), and this interaction accounted for 10.75% of the variation in diversity. In terms of the shape of the relationship between changes in the temperature regime and diversity, we found very little relationship between mean temperature and Hill's numbers at low levels of temporal variation (Figure 4); however, as temporal variation increased, lower mean temperatures were associated with increased diversity and lower diversity as mean temperature increased (Figure 4). In addition to changes in Hill's numbers, we also found that increased temporal variation was associated with reduced extinctions (*F*_(1, 30)_ = 10.46, *p* = .003, 21.58% variation explained, Figure 5a) and increased the probability a community contained predators increased (*p* = .047, 10.72% variation explained, Figure 5b). Neither the number of extinctions nor the probability of predators was affected by mean temperature, or an interaction between mean temperature and temperature variation (all *p* > .05).

## 4 DISCUSSION

A large portion of climate change research to date has focused on how mean changes to temperature will affect population size, persistence, and distribution of species of interest (Thornton et al., 2014). In recent years however, researchers have highlighted that climate change will not only affect mean temperatures but also levels of variation in temperature (Di Cecco & Gouhier, 2018). Here, we specifically compared how changes to mean temperature and temporal variation in temperature impact populations and communities in a long‐term (>10 generations) experiment. Our results indicate that changes in mean temperature as well as temporal variation in temperature can have significant effects at the population and community level. In general, we found that higher mean temperatures were associated with reduced population sizes, both higher and lower coefficients of variation of density through time, and reduced diversity when temporal variation in temperature was high. Conversely, temporal variation was associated with both increases and decreases in final density and reduced the number of observed extinctions, increased the probability a community contained predators.

Across multiple population measures, higher mean temperatures were associated with negative outcomes. Population sizes for four of the seven species were observed to be significantly lower at the end of the experiment when temperatures increased, and changes in temperature accounted for the majority of the variation in population size for five of our seven species. Increased temperatures are associated with increased metabolic demands for poikilothermic species (Sokolova & Lannig, 2008), potentially meaning fewer individuals can be supported for the same amount of resources, and that predators in our experiment may consume a greater number of their prey, explaining the reduced population sizes (Mccauley et al., 2015). Higher levels of temporal variation in temperature were associated with an increase in final densities for five of our seven species, often with steeper slopes than for changes in mean temperature; however, the amount of variation in density explained tended to be lower than for changes in mean temperature. High mean temperatures were also associated with both increases and decreases in population instability (i.e., population size CV). However, there appeared be no obvious taxonomic pattern to the directionality of these changes (for the two most closely related taxa, one showed increased stability, the other showed decreased), making it hard to deduce conclusions. Our results at the population level suggest that increased mean temperatures lead to smaller populations, while temporal variation in temperature in some cases partially mitigates this effect. However, we must point out that our experimental conditions do not replicate the full range of temperature, and fluctuations in temperature experienced by natural communities. The true relationship between components of the temperature regime and diversity is likely to be hump‐shaped (Kratina et al., 2017), as species will possess both upper and lower limits on their thermal tolerances.

At the community level, we found the highest levels of diversity when mean temperatures were low, and temporal variation was high. However, as temporal variation is reduced, these positive effects of low temperatures on diversity were lost. This reduction in diversity may be related to the reductions in population sizes associated with increased temperatures observed for four of our species. As Hill's numbers are a measure not only of the number of species but also the relative population sizes of those species (species evenness Roswell et al., 2021), reductions in some species may lead to significant changes in diversity by altering evenness, even in the absences of extinctions. The current study therefore highlights how population‐level changes can scale up to alterations in communities (Donohue et al., 2013). At low mean temperatures, the high diversity associated with increased variation may be a consequence of environmental variation reducing the impact of competitive dominants or increasing overall niche space (Shurin et al., 2010; Tilman, 1982), and the level of variation not being so high as to cause extreme conditions that lead to extinctions. However, at high mean temperatures when temporal variation was high, the positive effect of temporal variation on diversity was lost, suggesting that future scenarios in which mean temperatures increase and temporal variation is lost may generate reduced diversity.

While our study provides some insights into how changes in mean temperature and temperature variation affect diversity, the nature of the system and the length of the experiment mean several important processes are not accounted for. Among these, the design of our study does not account for how evolutionary changes in species may counteract or exacerbate the effects of changing temperature regime (Hoffmann & Sgró, 2011; Wang et al., 2021). Previous experiments have also demonstrated how global change‐mediated changes to trophic interactions can lead to altered diversity (Hammill, Johnson, et al., 2018), and how combined global changes can lead to reduced diversity (Atwood et al., 2015; Kratina et al., 2012; Tabi et al., 2019). While we are looking at two components of temperature regime in the current experiment, we are only looking at one component of global change (temperature). This focus on temperature overlooks multiple other changes such as nutrient levels, and how these lead to changes in diversity. While the current study identifies several population‐ and community‐level changes following altered temperature regimes, combining these temperature experiments would increase the realism of the experiments.

Aquatic microcosms can be used to test a suite of population‐ and community‐level processes (Altermatt et al., 2015); however, the artificial nature of the system impacts overall applicability. While the food web utilized in our experiment contains multiple trophic levels and competition, this artificial community remains very simple compared to natural systems (Srivastava et al., 2004). The ecological simplicity of our artificial microcosms means that although we can observe diversity changes with a high degree of precision, they do lack a level of accuracy, and the results may not directly translate to natural systems.

Across all the different population and community metrics we measured, it would appear that increased mean temperatures had generally negative effects, reducing population sizes and stability. However, increased temporal variation had several positive effects, including increasing the chance a community contained predators, increasing diversity at low mean temperatures, and reducing extinctions. Our results are somewhat concerning given that climate projections indicate not only increased temperatures, but potentially reductions in temporal variation (Di Cecco & Gouhier, 2018). In the case of changes in final population densities, the amount of variation associated with alterations in mean temperature was greater than for temporal variation in temperature. This result suggests that even if temporal variation in temperature increased with climate change, its positive effects may not be sufficient to counteract the negative impacts of increased mean temperatures. Future climate predictions, coupled with our results, therefore, suggest that the stabilizing effect of temporal variation may not be able mitigate some of the ecological impacts associated with increased temperatures.

## CONFLICT OF INTEREST

The authors declare they have no conflicts of interest.

## AUTHOR CONTRIBUTIONS

**Edd Hammill:** Conceptualization (equal); Methodology (equal); Writing – original draft (lead); Writing – review & editing (lead). **Riley Dart:** Methodology (equal); Project administration (equal); Writing – review & editing (supporting).

## Data Availability

All data used in this MS have been uploaded to Dryad https://doi.org/10.5061/dryad.7m0cfxpwh.
